# Anti-Inflammatory Effects of Rhamnetin on Bradykinin-Induced Matrix Metalloproteinase-9 Expression and Cell Migration in Rat Brain Astrocytes

**DOI:** 10.3390/ijms23020609

**Published:** 2022-01-06

**Authors:** Chien-Chung Yang, Li-Der Hsiao, Ya-Fang Shih, Zih-Yao Yu, Chuen-Mao Yang

**Affiliations:** 1 Department of Traditional Chinese Medicine, Chang Gung Memorial Hospital, Kwei-San, Tao-Yuan 33302, Taiwan; r55161@cgmh.org.tw; 2 School of Traditional Chinese Medicine, College of Medicine, Chang Gung University, Kwei-San, Tao-Yuan 33302, Taiwan; 3 Department of Pharmacology, College of Medicine, China Medical University, Taichung 40402, Taiwan; lidesiao@livemail.tw (L.-D.H.); shihyafang@mail.cmu.edu.tw (Y.-F.S.); zyy0427@tmu.edu.tw (Z.-Y.Y.); 4 Ph.D. Program for Biotech Pharmaceutical Industry, China Medical University, Taichung 40402, Taiwan; 5 Department of Post-Baccalaureate Veterinary Medicine, College of Medical and Health Science, Asia University, Wufeng, Taichung 41354, Taiwan

**Keywords:** neuroinflammation, rhamnetin, NADPH oxidase, ROS, BK, MMP-9, astrocytes

## Abstract

Bradykinin (BK) has been shown to induce matrix metalloproteinase (MMP)-9 expression and participate in neuroinflammation. The BK/MMP-9 axis can be a target for managing neuroinflammation. Our previous reports have indicated that reactive oxygen species (ROS)-mediated nuclear factor-kappaB (NF-κB) activity is involved in BK-induced MMP-9 expression in rat brain astrocytes (RBA-1). Rhamnetin (RNT), a flavonoid compound, possesses antioxidant and anti-inflammatory effects. Thus, we proposed RNT could attenuate BK-induced response in RBA-1. This study aims to approach mechanisms underlying RNT regulating BK-stimulated MMP-9 expression, especially ROS and NF-κB. We used pharmacological inhibitors and siRNAs to dissect molecular mechanisms. Western blotting and gelatin zymography were used to evaluate protein and MMP-9 expression. Real-time PCR was used for gene expression. Wound healing assay was applied for cell migration. 2ʹ,7ʹ-dichlorodihydrofluorescein diacetate (H2DCF-DA) and nicotinamide adenine dinucleotide phosphate (NADPH) oxidase (NOX) were used for ROS generation and NOX activity, respectively. Promoter luciferase assay and chromatin immunoprecipitation (ChIP) assay were applied to detect gene transcription. Our results showed that RNT inhibits BK-induced MMP-9 protein and mRNA expression, promoter activity, and cell migration in RBA-1 cells. Besides, the levels of phospho-PKCδ, NOX activity, ROS, phospho-ERK1/2, phospho-p65, and NF-κB p65 binding to MMP-9 promoter were attenuated by RNT. In summary, RNT attenuates BK-enhanced MMP-9 upregulation through inhibiting PKCδ/NOX/ROS/ERK1/2-dependent NF-κB activity in RBA-1.

## 1. Introduction

In the central nervous system (CNS), astrocytes are a type of glial cell that play a pivotal role in brain homeostasis and the pathogenesis of brain diseases. In particular, they participate in regulating brain inflammation. However, it is intriguing as astrocytes have been known to play dual roles either by progressing or suppressing inflammation. For instance, the early stage of migration is beneficial as it provides support to the site of injury by surrounding and preventing the infiltration of pro-inflammatory factors into the injury sites [1]. In contrast, prolonged astrocyte migration or proliferation, also known as astrogliosis, is detrimental and irreversible as it causes astrocytes to release more pro-inflammatory molecules, such as extracellular matrix (ECM) and cytokines, which hence advances the severity of the brain injury [2].

Bradykinin (BK) is a short-lived peptide that belongs to the kinins family, which is involved in the etiology of several brain inflammatory disorders, such as brain edema, traumatic brain injury, and ischemic and hemorrhagic stroke [3,4,5,6]. It has been disclosed that BK concentration is significantly increased under these insults. Our previous studies have revealed that BK could induce matrix metalloproteinase (MMP)-9 upregulation in astrocytes [7,8,9,10]. Our reports suggested that BK-stimulated astrocytes contribute inflammatory reaction leading to neuronal apoptosis via upregulation of MMP-9, heme oxygenase-1 (HO-1)/carbon monoxide (CO) axis, and reactive oxygen species (ROS) [7,8,9,11]. In these studies, we have found that BK-induced MMP-9 expression was mediated through protein kinase C(PKC)δ-extracellular regulated kinase (ERK)1/2 and nuclear factor-kappaB (NF-κB) pathway in rat brain astrocytes (RBA-1) [7,8]. MMPs belong to the calcium- and zinc-dependent endopeptidases which are involved in the pathophysiological process by degrading the ECM, leading to the breakdown of the blood–brain barrier (BBB) and chemotaxis of inflammatory cells. Among MMPs, MMP-9 especially possesses a crucial role in regulating the cellular reactions to inflammation. Increased expression and activity of MMP-9 are observed when the brain suffers from injury or diseases, such as cerebral ischemia, stroke, Alzheimer’s disease, or cancer/tumor metastasis [12,13,14]. Thus, exploration of compounds with the property of targeting BK/MMP-9 system could be new therapeutic candidates to manage neuroinflammation-related brain diseases.

Flavonoids, a class of polyphenolic compounds, have been known to prevent inflammation, including downregulation of MMP-9 expression [15,16]. Several lines of evidence have shown the beneficial effects of flavonoids in the treatment of inflammatory diseases which are attributed to targeting MMP-9 [17,18,19]. In addition, flavonoids are also known to have their effects on balancing the level of oxidative stress during inflammation. Rhamnetin (RNT) is one kind of flavonoid that has been demonstrated to be an effective therapeutic agent in many kinds of diseases related to inflammation, including cancer [20], ischemia [21], and traumatic brain injury [22]. A growing body of evidence has indicated that RNT or its derivatives possess anti-inflammatory effects via protecting against oxidative stress, targeting MMPs, and regulating the activation of protein kinases and transcription factors [23,24,25,26]. However, in RBA-1 cells, whether RNT could inhibit MMP-9 expression induced by BK is still elusive. The present study aims to dissect the molecular mechanisms by which RNT mitigates BK-induced responses in RBA-1 cells, including MMP-9 expression and cell migration. We evaluated the roles of nicotinamide adenine dinucleotide phosphate (NADPH) oxidase (NOX)/ROS, and protein kinases, such as ERK1/2, PKCδ and transcription factor NF-κB, in the MMP-9 expression and cell migration induced by BK. The inhibitory effects of RNT on BK-mediated responses were assessed. The present results revealed that RNT exerts anti-inflammatory properties, at least in part, through inhibiting PKCδ/NOX/ROS/ERK1/2 signaling cascade-dependent NF-κB activation in RBA-1 cells. Thus, RNT could be beneficial in the treatment of brain inflammation.

## 2. Results

### 2.1. RNT Inhibited BK-Induced MMP-9 Expression and Cell Migration in RBA-1 Cells

**Figure 1:**
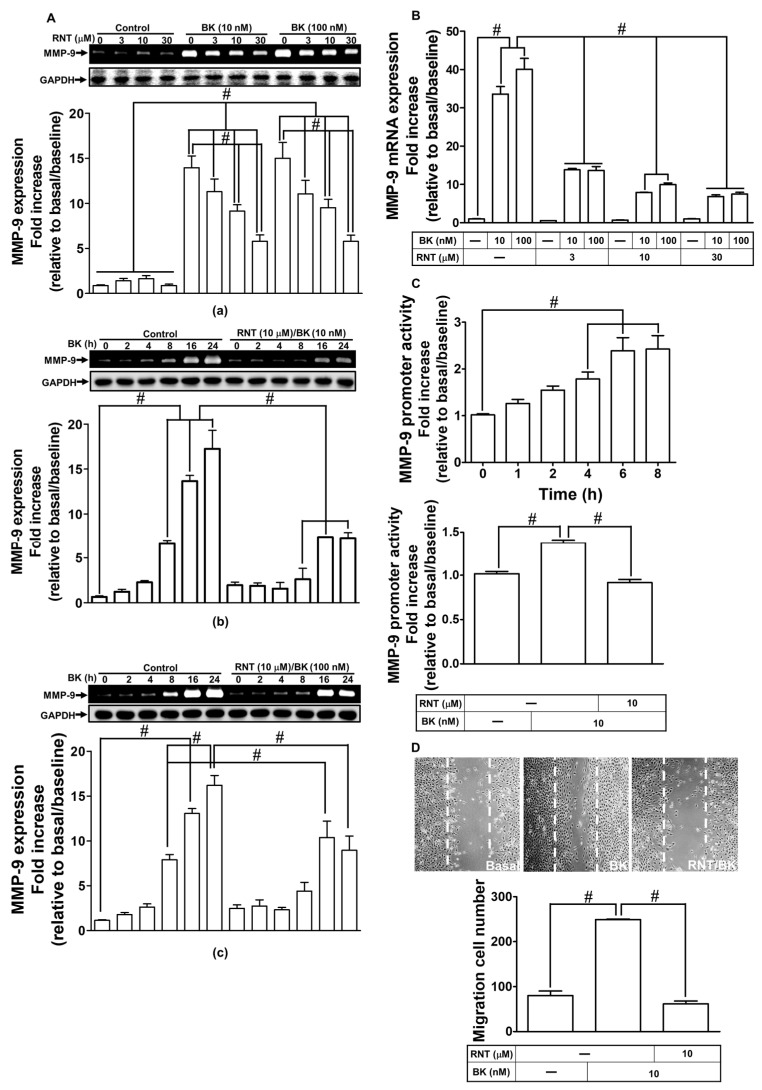
Rhamnetin (RNT) attenuates MMP-9 expression and cell migration induced by bradykinin (BK) in RBA-1 cells. (**A**) The cells were incubated with various concentrations of RNT (3, 10, 30 μM) for 1 h and then challenged with various doses of BK (10, 100 nM) for 24 h (**a**). The cells were incubated with 10 μM RNT for 1 h and then challenged with (**b**) 10 nM and (**c**) 100 nM of BK for 2, 4, 8, 16, and 24 h. Media and cell lysates were collected to determine MMP-9 level and GAPDH, by gelatin zymography and Western blot, respectively. (**B**) Cells were pretreated without or with RNT (3, 10, 30 μM) for 1 h and then incubated with BK (10, 100 nM) for 16 h. The *mmp-9* gene expression was determined by real-time PCR. (**C**) Cells were incubated with 10 nM BK for 1, 2, 4, 6, and 8 h (upper panel). Cells were pretreated without or with 10 μM RNT for 1 h and then incubated with 10 nM BK for 6 h (lower panel). The MMP-9 promoter activity was determined by a luciferase reporter assay. (**D**) Cells were pretreated without or with RNT (10 μM) for 1 h and then incubated with BK (10 nM) for 48 h. The number of migratory cells was determined (magnification = 40×). Data are expressed as mean ± SEM of three independent experiments. # *p* < 0.01 as compared with the cells exposed to vehicle or BK, as indicated. Abbreviations: BK, bradykinin; RNT, rhamnetin; GAPDH, glyceraldehyde 3-phosphate dehydrogenase.

MMPs are involved in the pathogenesis of neuronal cell death and several brain diseases [27]. In RBA-1 cells, BK has been shown to induce MMP-9 expression [7]. According to a previous study, it was demonstrated that RNT might have a strong neuroprotective potential mediated through inhibiting both MMP-9 and oxidative stress in brain insults [26]. Thus, we investigated whether, in RBA-1 cells, RNT inhibits BK-induced MMP-9 expression. As presented in Figure 1A, in RBA-1 cells, pretreatment with various concentrations (3, 10, and 30 μΜ) of RNT can dose-dependently attenuate BK (10, 100 nM)-induced MMP-9 expression (Figure 1A-a; *p* < 0.0001), as well as RNT (10 μΜ) time-dependently inhibited BK (10, 100 nM)-induced MMP-9 expression (Figure 1A-b,c; *p* < 0.0001) examined by gelatin zymography. To investigate whether RNT inhibits BK response via a transcription level, we further examined promoter activity and gene expression of MMP-9 by luciferase reporter assay and real-time PCR, respectively. Our data showed that RNT dose-dependently reduced BK-induced mRNA expression of MMP-9 (Figure 1B; *p* < 0.0001). In addition, we also revealed that the promoter activity of MMP-9 was boosted upon BK exposure within 4–8 h of the observation period (*p* < 0.0001), which was impeded by 10 μM RNT pretreatment (Figure 1C; *p* < 0.0001). Finally, we found that exposure of 10 nM BK induced cell migration in RBA-1 cells which can be blocked by pretreatment of 10 μM RNT measured by cell migration assay (Figure 1D; *p* < 0.0001). These results suggested that, in RBA-1 cells, RNT exerts inhibitory effects on MMP-9 expression and cell migration stimulated by BK.

### 2.2. The Inhibitory Effects of RNT on BK-Induced MMP-9 Expression via Suppressing PKCδ

**Figure 2:**
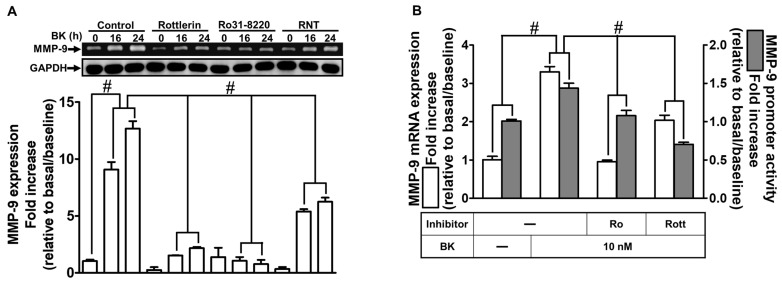

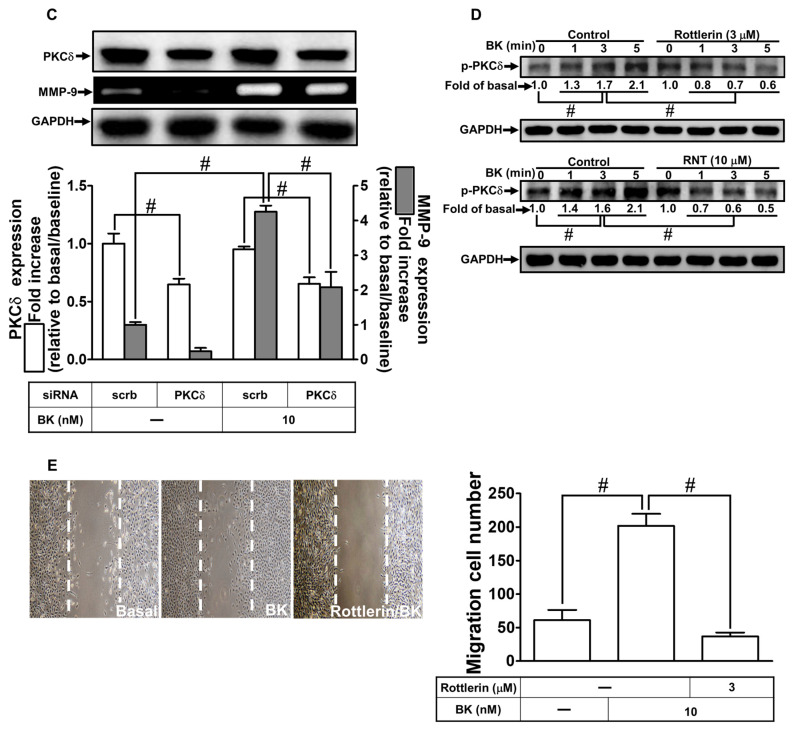
RNT attenuates MMP-9 expression and cell migration induced by BK via regulating PKCδ signaling in RBA-1 cells. (**A**) Cells were pretreated with 3 μM rottlerin, 10 μM Ro31-8220, or 10 μM RNT for 1 h and then stimulated with BK (10 nM) for 16 and 24 h. The levels of MMP-9 in the medium and GAPDH in cell lysates were examined by gelatin zymography and Western blot, respectively. (**B**) Cells were pretreated with 3 μM rottlerin or 10 μM Ro31-8220 for 1 h and then incubated with BK (10 nM) of 6 h for analyzing MMP-9 mRNA expression and promoter activity, respectively, determined by real-time PCR and promoter assay. (**C**) Cells were individually transfected with scrambled (Scrb) or PKCδ siRNA and then incubated with BK (10 nM) for 24 h. The media and cell lysates were collected to determine the levels of MMP-9 by gelatin zymography and the levels of GAPDH and PKCδ by Western blot, respectively. (**D**) Cells were individually pretreated without or with rottlerin (3 μΜ) or RNT (10 μM) for 1 h and then challenged with BK (10 nM) for the indicated time intervals (1, 3, and 5 min). The phosphorylation of PKCδ was determined by Western blot with GAPDH as loading controls. (**E**) Cells were pretreated without or with 3 μM rottlerin for 1 h and then incubated with BK (10 nM) for 48 h. The number of migratory cells was determined (magnification = 40×). Data are expressed as mean ± SEM of three independent experiments. # *p* < 0.01 as compared with the cells exposed to vehicle or BK, as indicated.

Previous reports have indicated that PKCs are involved in BK-induced inflammatory responses such as MMP-9 upregulation [6,28,29]. To clarify whether BK-induced MMP-9 expression is mediated through PKCs, which is inhibited by RNT via blocking PKC activity, we used RNT, Rottlerin (selective PKCδ inhibitor), and Ro31-8220 (non-selective PKC inhibitor). As presented in Figure 2A, pretreatment with RNT or these PKC inhibitors time-dependently reduced BK-induced MMP-9 expression determined by gelatin zymography during the period of observation (*p* < 0.0001). Additionally, pretreatment with Rottlerin or Ro31-8220 significantly attenuated BK-induced promoter activity (*p* < 0.0001) and mRNA level (*p* < 0.0001) of MMP-9, respectively examined by luciferase reporter assay and real-time PCR (Figure 2B). To further reveal the effect of PKCδ on MMP-9 upregulation induced by BK in RBA-1 cells, we knocked down the PKCδ level by transfection with PKCδ siRNA (*p* = 0.0043), which downregulated MMP-9 expression (*p* < 0.0001) induced by BK as well (Figure 2C). Besides, our data demonstrated that pretreatment with Rottlerin abrogated BK-stimulated phosphorylation of PKCδ (Figure 2D; *p* < 0.0001). To further determine whether RNT interrupted the PKCδ pathway to inhibit BK-induced MMP-9 expression, pretreatment of RBA-1 cells with 10 μM RNT reduced phosphorylation of PKCδ stimulated by BK during the period of observation (Figure 2D; *p* < 0.0001). Further, as shown in Figure 2E, these data demonstrated that BK-induced cell migration was inhibited by Rottlerin (*p* = 0.0003). These results suggested that BK activates the PKCδ pathway, leading to MMP-9 upregulation and cell migration which were inhibited by RNT in RBA-1 cells.

### 2.3. NOXs Participate in BK-Induced MMP-9 Expression and Cell Migration

**Figure 3:**
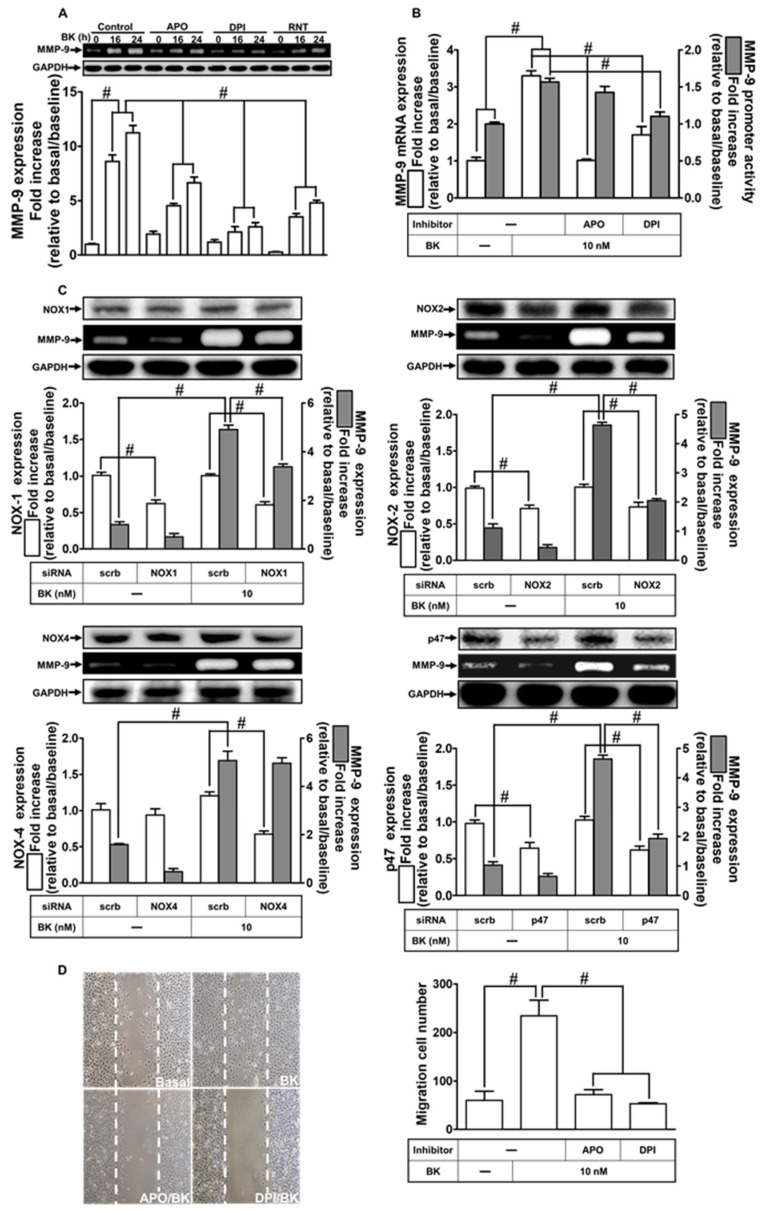
RNT inhibits nicotinamide adenine dinucleotide phosphate (NADPH) oxidase (NOX)-dependent MMP-9 expression and cell migration induced by BK in RBA-1 cells. (**A**) Cells were pretreated with 100 μM apocynin (APO), 10 μM diphenyleneiodonium (DPI), or 10 μM RNT for 1 h and then stimulated with BK (10 nM) for 16 and 24 h. The levels of MMP-9 in the media and GAPDH in cell lysates were examined by gelatin zymography and Western blot, respectively. (**B**) Cells were pretreated with 100 μM APO or 10 μM DPI for 1 h and then incubated with BK (10 nM) of 6 h for analyzing MMP-9 mRNA expression and promoter activity, respectively, determined by real-time PCR and promoter assay. (**C**) Cells were individually transfected with scrambled (Scrb), NOX1, NOX2, NOX4, or p47^phox^ siRNA and then incubated with BK (10 nM) for 24 h. The media and cell lysates were collected to determine the levels of MMP-9 by gelatin zymography and the levels of GAPDH, NOX1, NOX2, NOX4, and p47^phox^ proteins by Western blot, respectively. (**D**) Cells were pretreated without or with 100 μM APO or 10 μM DPI for 1 h and then incubated with BK (10 nM) for 48 h. The number of cell migration was determined (magnification = 40×). Data are expressed as mean ± SEM of three independent experiments. # *p* < 0.01 as compared with the cells exposed to vehicle or BK, as indicated. Abbreviations: APO, apocynin; DPI, diphenyleneiodonium.

NOXs have been presented in various types of cells, which have a vital role in inflammation, including MMP-9 upregulation [30,31]. Our previous study has shown that NOX2 is regulated by upstream signaling PKCα engaged in MMP-9 expression and induced by BK [9]. Here, we aimed to explore whether other isoforms of NOXs are involved in BK-induced responses, including MMP-9 expression. First, p47 inhibitor apocynin (APO) and NOX inhibitor diphenyleneiodonium (DPI) were used to reveal the roles of NOXs in BK-induced reactions. As presented in Figure 3A, BK-enhanced MMP-9 expression was abolished by either APO or DPI, and also RNT (*p* < 0.0001). In addition, APO or DPI also attenuated BK-stimulated MMP-9 promoter activity (*p* < 0.0001) and mRNA level (*p* < 0.0001) examined by luciferase reporter assay and real-time PCR, respectively (Figure 3B). Further, to differentiate whether NOX1, NOX2, NOX4, and subunit p47^phox^ are involved in MMP-9 expression, RBA-1 cells were transfected with these respective siRNAs. Our data showed that downregulation of NOX1, NOX2, or p47^phox^ protein expression (*p* < 0.0004) also reduced MMP-9 upregulation by BK (*p* < 0.0001) except downregulation of NOX4 in these cells (Figure 3C). Finally, pretreatment with APO or DPI abrogated BK-induced cell migration (Figure 3D; *p* = 0.0006). These results indicated that NOX1, NOX2, and p47^phox^ signaling components participated in the upregulation of MMP-9 and cell migration in RBA-1 cells stimulated by BK.

### 2.4. RNT Attenuates BK-Induced MMP-9 Expression via Reducing ROS Generation

**Figure 4:**
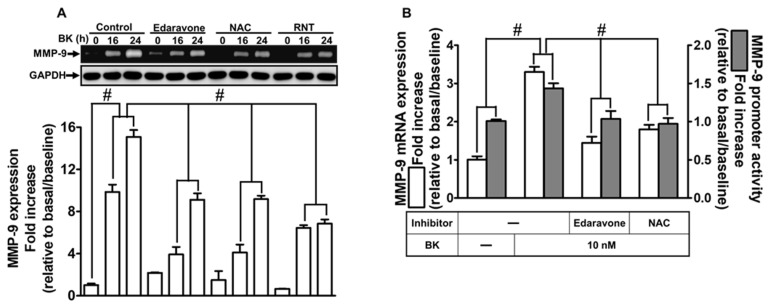

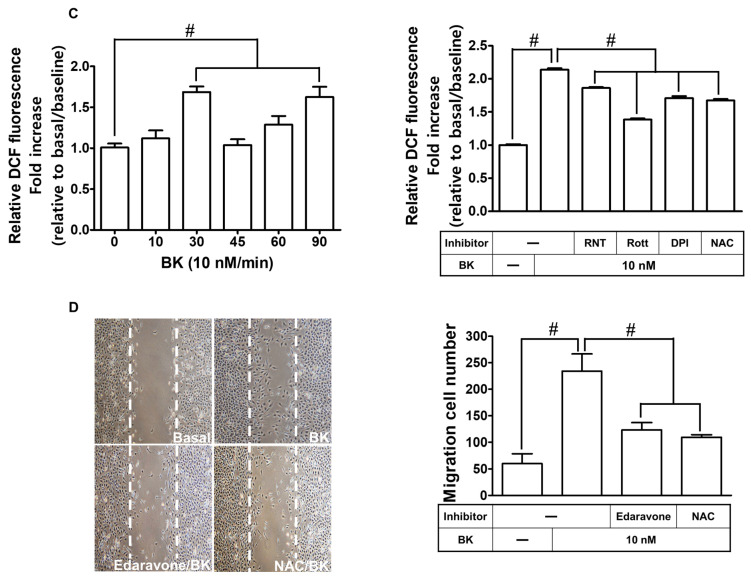
RNT mitigates reactive oxygen species (ROS) generation to block BK-induced MMP-9 expression and cell migration in RBA-1 cells. (**A**) Cells were pretreated with 30 μM edaravone, 1 mM N-acetylcysteine (NAC), or 10 μM RNT for 1 h and then stimulated with BK (10 nM) for 16 and 24 h. The levels of MMP-9 in the media and GAPDH in cell lysates were examined by gelatin zymography and Western blot, respectively. (**B**) Cells were pretreated with 30 μM edaravone or 1 mM NAC for 1 h and then incubated with BK (10 nM) of 6 h for analyzing MMP-9 mRNA expression and promoter activity, respectively, determined by real-time PCR and promoter assay. (**C**) Cells were incubated with BK (10 nM) for the indicated time intervals (10, 30, 45, 60, and 90 min; left panel), and cells were pretreated without or with 3 μM rottlerin, 10 μM DPI, 1 mM NAC, or 10 μM RNT for 1 h and then incubated with 10 nM BK for 30 min (right panel). The fluorescence intensity of DCFH-DA staining was detected using a fluorescence microplate reader (**D**) Cells were pretreated without or with 30 μM edaravone or 1 mM NAC for 1 h and then incubated with BK (10 nM) for 48 h. The number of cell migration was determined (magnification = 40×). Data are expressed as mean ± SEM of three independent experiments. # *p* < 0.01 as compared with the cells exposed to vehicle or BK, as indicated. Abbreviations: NAC, N-acetylcysteine; ROS, reactive oxygen species.

Multiple data sources have indicated that ROS participate in MMP-9 expression induced by various stimuli [30,31]. We also found that NOX-2-dependent ROS engage in BK-stimulated MMP-9 expression. RNT has been demonstrated as a member of flavonoids protecting against oxidative stress [22,26]. Here, we explored whether RNT reduced BK-induced MMP-9 upregulation by inhibiting ROS generation. First, we used two ROS scavengers, edaravone and NAC, to reveal whether BK was mediated through ROS signaling to induce MMP-9 expression. As shown in Figure 4A, in RBA-1 cells, pretreatment with edaravone, NAC, or RNT can significantly decrease MMP-9 expression induced by BK (*p* < 0.0001). Correspondingly, edaravone or NAC also inhibited MMP-9 promoter activity (*p* < 0.0001) and mRNA level (*p* < 0.0001) investigated by luciferase reporter assay and real-time PCR, respectively (Figure 4B). Next, we evaluated the intracellular ROS level upon BK exposure using H_2_DCFDA staining. As shown in Figure 4C, intracellular ROS level was increased within 30 min of BK challenge and then appeared a secondary peak of generation of ROS within 90 min (*p* < 0.0001). Further, we found that pretreatment with DPI, NAC, RNT, or inhibitor of PKCδ (Rottlerin) can reduce ROS generation after 30 min BK exposure (*p* < 0.0001). Additionally, our data also demonstrated that edaravone or NAC pretreatment blocked BK-induced cell migration in RBA-1 cells (Figure 4D; *p* = 0.0004). On the basis of the above findings, we suggested that ROS generation engages in MMP-9 expression and cell migration in RBA-1 cells triggered by BK. Moreover, RNT attenuates BK-induced responses, at least in part, by blocking the intracellular ROS level.

### 2.5. RNT Attenuates BK-Induced MMP-9 Expression Mediated via Suppressing ERK1/2

**Figure 5:**
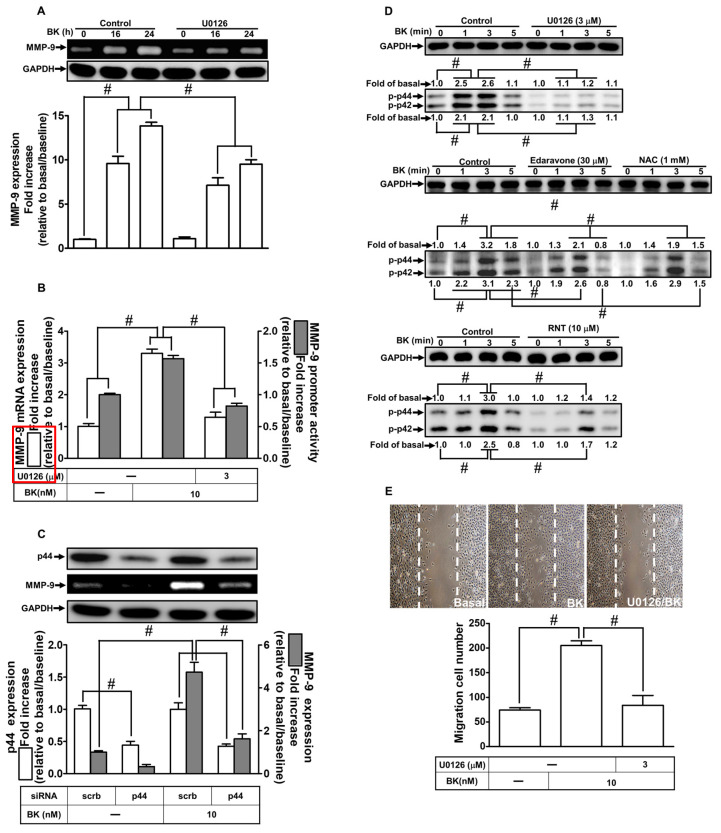
RNT attenuates p44/p42 activity induced by BK in RBA-1 cells. (**A**) Cells were pretreated with 3 μM U0126 for 1 h and then stimulated with BK (10 nM) for 16 and 24 h. The levels of MMP-9 in the media and GAPDH in cell lysates were examined by gelatin zymography and Western blot, respectively. (**B**) Cells were pretreated with 3 μM U0126 for 1 h and then incubated with BK (10 nM) of 6 h for analyzing MMP-9 mRNA expression and promoter activity, respectively, determined by real-time PCR and promoter assay. (**C**) Cells were individually transfected with scrambled (Scrb) or p44 siRNA and then incubated with BK (10 nM) for 24 h. The media and cell lysates were collected to determine the levels of MMP-9 by gelatin zymography and the levels of GAPDH and p44 proteins by Western blot, respectively. (**D**) Cells were individually pretreated without or with U0126 (3 μΜ), edaravone (30 μM), NAC (1 mM), or RNT (10 μM) for 1 h and then challenged with BK (10 nM) for the indicated time intervals (1, 3, and 5 min). The phosphorylation of p44/p42 was determined by Western blot with GAPDH as loading controls. (**E**) Cells were pretreated without or with 3 μM U0126 for 1 h and then incubated with BK (10 nM) for 48 h. The number of migratory cells was determined (magnification = 40×). Data are expressed as mean ± SEM of three independent experiments. # *p* < 0.01 as compared with the cells exposed to vehicle or BK, as indicated.

ERK1/2 signaling has been implicated in various pathophysiological conditions in different types of cells, including MMP-9 upregulation and cell migration [30,32]. Our previous reports have also discovered that in RBA-1 cells, ERK1/2 is a crucial signaling pathway in BK-induced MMP-9 upregulation [8]. Herein, we evaluated whether RNT mediated through regulating ERK1/2 activity to reduce BK-induced responses. First, in RBA-1 cells, we adopted U0126 (a MEK1/2 inhibitor) to dissect the involvement of ERK1/2 in BK-induced MMP-9 expression and cell migration. Pretreatment with 3 μM U0126 time-dependently inhibited the BK-stimulated MMP-9 expression examined by gelatin zymography (Figure 5A; *p* < 0.0001). Further, pretreatment with U0126 also reduced BK-stimulated MMP-9 promoter activity (*p* < 0.0001) and mRNA (*p* = 0.0006) expression detected by luciferase reporter assay and real-time PCR, respectively (Figure 5B). In addition, we further verified the function of ERK1/2 in BK-induced upregulation of MMP-9 using the approach of p44 siRNA transfection. The presented data revealed that downregulation of p44 protein by using p44 siRNA transfection (*p* < 0.0001) also attenuated the level of MMP-9 (*p* < 0.0001) stimulated by BK (Figure 5C). Further, we found that the levels of phosphorylation of ERK1/2 were enhanced upon BK challenge (*p* < 0.0001), which was attenuated by pretreatment with U0126 (*p* < 0.0001) during the period of observation (Figure 5D). Moreover, our experiments also demonstrated that pretreatment with RNT (*p* < 0.0001), edaravone (*p* < 0.0001), or NAC (*p* < 0.0001) also inhibited the increase in BK-induced phosphorylation of ERK1/2, suggesting that ERK1/2 is a downstream signaling pathway of ROS. In addition, RNT could attenuate ERK1/2 activity in RBA-1 cells stimulated by BK. Finally, we observed that pretreatment of RBA-1 cells with U0126 (3 μM) for 1 h and then exposed the cells with BK for 48 h to investigate the functional role of ERK1/2 in cell migration. Images in Figure 5E showed that U0126 abrogated cell migration induced by BK (*p* < 0.0001). These results implied that ERK1/2 participates in BK-induced MMP-9 upregulation and cell migration. The inhibitory effect of RNT on BK-induced response was, at least partially, mediated through suppressing ERK1/2 activity.

### 2.6. RNT Inhibits BK-Induced MMP-9 Expression via Attenuating NF-κB Activation

**Figure 6:**
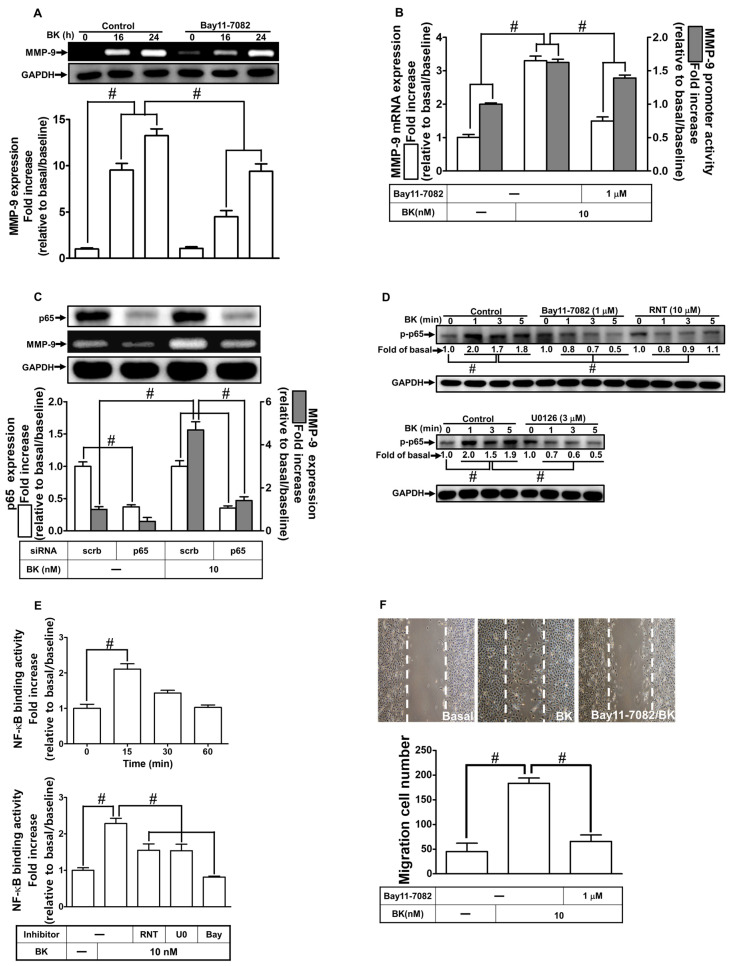
RNT blocks NF-κB activity and promoter binding ability induced by BK in RBA-1 cells. (**A**) Cells were pretreated with 1 μM Bay11-7082 for 1 h and then stimulated with BK (10 nM) for 16 and 24 h. The levels of MMP-9 in the media and GAPDH in cell lysates were examined by gelatin zymography and Western blot, respectively. (**B**) Cells were pretreated with 1 μM Bay11-7082 for 1 h and then incubated with BK (10 nM) of 6 h for analyzing MMP-9 mRNA expression and promoter activity, respectively, by real-time PCR and promoter assay. (**C**) Cells were individually transfected with scrambled (Scrb) or p65 siRNA and then incubated with BK (10 nM) for 24 h. The media and cell lysates were collected to determine the levels of MMP-9 by gelatin zymography and the levels of GAPDH and p65 by Western blot, respectively. (**D**) Cells were individually pretreated without or with inhibitors of Bay11-7082(1 μM), U0126 (3 μΜ), or RNT (10 μM) for 1 h, respectively, and then challenged with BK (10 nM) for the indicated time intervals (1, 3, and 5 min). The phosphorylation of p65 was determined by Western blot with GAPDH as loading controls. (**E**) Cells were stimulated with BK (10 nM) for the indicated intervals (15, 30, and 60 min, upper panel), and cells were pretreated without or with Bay11-7082 (1 μΜ), U0126 (3 μΜ), or RNT (10 μM) for 1 h and then challenged with BK (10 nM) for 15 min (low panel). The levels of NF-κB p65 binding with the promoter of MMP-9 were determined by chromatin immunoprecipitation (ChIP) assay. (**F**) Cells were pretreated without or with 1 μM Bay11-7082 for 1 h and then incubated with BK (10 nM) for 48 h. The number of migratory cells was determined (magnification = 40×). Data are expressed as mean ± SEM of three independent experiments. # *p* < 0.01 as compared with the cells exposed to vehicle or BK, as indicated.

NF-κB is activated and involved in many human diseases, including the events of cerebral inflammatory responses [33]. Our previous reports have discovered that many pro-inflammatory mediators can upregulate MMP-9 levels mediated through NF-κB activation in various types of cells [30,32]. An earlier report performed in RBA-1 cells also indicated that NF-κB has an essential function in BK-induced MMP-9 expression [8]. In addition, the effect of RNT on NF-κB is still elusive. First, to assess whether NF-κB engages in BK-induced upregulation of MMP-9, we adopted Bay11-7082, an NF-κB inhibitor, for this purpose. The BK-induced upregulation of the MMP-9 level was reduced by Bay11-7082 pretreatment (Figure 6A; *p* < 0.0001). Further, Bay11-7082 pretreatment also attenuated the BK-induced MMP-9 promoter activity (*p* < 0.0001) and mRNA level (*p* < 0.0001) indicated by luciferase reporter assay and real-time PCR, respectively (Figure 6B). Our experiments used specific p65 siRNA to knock down p65 and to ascertain the above findings. Transfection with p65 siRNA knocked down the levels of p65 (*p* < 0.0001) and also BK-stimulated MMP-9 expression (Figure 6C; *p* < 0.0001). Next, in RBA-1 cells, we explored whether phosphorylation of p65 participates in BK-induced response. Data in Figure 6D showed that BK can induce increased levels of phospho-NF-κB p65 in a time-dependent manner (*p* < 0.0001). In addition, pretreatment with 10 μM RNT (*p* < 0.0001), 3 μM U0126 (*p* < 0.0001), or 1 μM Bay11-7082 (*p* < 0.0001) each could inhibit the phosphorylation of NF-κB p65. Further, we investigated whether activated NF-κB p65 could bind with the promoter of MMP-9 leading to gene transcription, determined by a ChIP assay. As shown in Figure 6E, BK can enhance the binding ability of NF-κB p65 with the promoter of MMP-9 within 30 min in RBA-1 cells (*p* < 0.0001), which was blocked by pretreatment with 10 μM RNT, 3 μM U0126, or 1 μM Bay11-7082 (*p* = 0.0001). Moreover, to investigate the function of NF-κB in cell migration, RBA-1 cells were pretreated without or with 1 μM Bay11-7082 for 1 h and then exposed to BK for 48 h. Images in Figure 6F showed that Bay11-7082 abrogated cell migration stimulated by BK (*p* < 0.0001). These findings implied that in RBA-1 cells, NF-κB activity regulated by the ERK1/2 signaling pathway plays a critical role in MMP-9 expression and cell migration stimulated by BK. Besides, RNT inhibits BK-induced responses, at least partially, via inhibiting NF-κB activity in these cells.

## 3. Discussion

**Figure 7:**
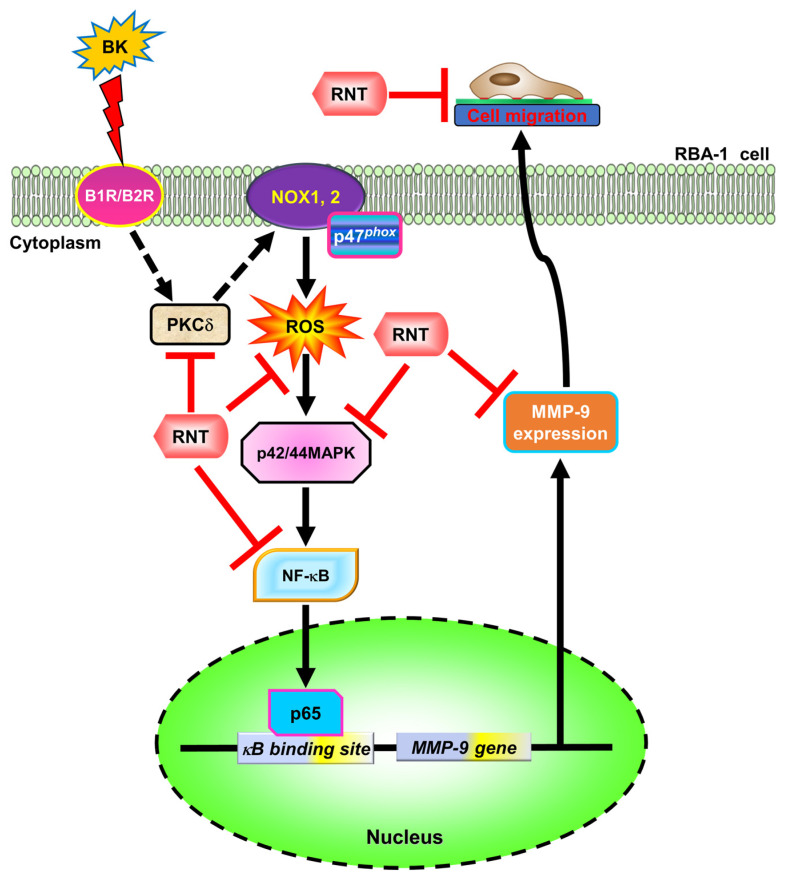
The schematic signaling pathways contribute to the effects of RNT on the BK-induced MMP-9 expression in RBA-1 cells. RNT blocks PKCδ/NADPH oxidase/ROS generation/p44/p42-dependent NF-κB p65 binding with MMP-9 promoter induced by BK, leading to attenuating MMP-9 expression and cell migration of RBA-1 cells. “→” means “activated”; “⊥” means “inhibited”.

MMP-9 possesses pivotal functions in the physiology of brain development and the pathogenesis of neuroinflammation [34]. Upregulation of MMP-9 has been well known to cause BBB breakdown and recruitment of inflammatory cells [35] related to the pathologic processes of many brain insults, such as Alzheimer’s disease, stroke, and tumor metastasis [12,13,14]. BK is also highly associated with brain inflammatory diseases, such as stroke, brain edema, and traumatic brain injury [3,5,6]. Therefore, exploration of potential compounds targeting the BK/MMP-9 axis and their mechanisms are warranted. Our previous studies have consistently demonstrated the involvement of MMP-9 in inflammation upon exposure to various pro-inflammatory mediators, such as tumor necrosis factor (TNF)-α [36], interleukin-1β (IL-1β) [37], thrombin [38], and lipopolysaccharides (LPS) [39]. In the past decade, we have discovered that the MMP-9 upregulation induced by BK contributes to the inflammatory state in astrocytes [8]. Some studies demonstrated the anti-inflammatory effects of RNT in various brain diseases such as traumatic brain injury [22], stroke [26], and alcohol addiction [40]. However, the detailed mechanisms of RNT are still uncertain in inflammatory brain insults, albeit it has been shown to have the beneficial effects. In the present study, we established the mechanisms by which, in rat brain astrocytes, RNT inhibits the BK-induced MMP-9 upregulation and cell migration. We revealed that RNT could attenuate BK-enhanced MMP-9 expression and cell migration through, at least partially, inhibition of PKCδ/NOX/ROS/ERK1/2-dependent NF-κB activation in RBA-1 cells (Figure 7).

PKCs are a family of protein kinases that regulate numerous cellular processes via phosphorylation of serine and threonine residues on many target proteins. Their importance has been well documented in neuroinflammatory responses [41,42]. PKCs signaling has revealed the engagement in the regulation of MMP-9 expression induced by many proinflammatory mediators, such as thrombin [43], neurotensin [44], and 12-O-tetradecanoylphorbol-13-acetate (TPA) [45]. Our previous report indicated that BK enhances MMP-9 expression through activation of PKCδ [7]. Another previous report also indicated that RNT could inhibit the PKC pathway [46]. Additionally, our recent study demonstrated that galangin suppresses MMP-9 expression induced by thrombin in SK-N-SH cells through inhibition of PKCα/β/δ [32]. In line with these studies, our results revealed that the MMP-9 expression and cell migration induced by BK are attenuated by RNT, which is mediated through inhibition of PKCδ activation in RBA-1 cells.

The excessive generation of NOX-derived ROS is related to many degenerative diseases, such as atherosclerosis [47] and Alzheimer’s disease [48]. NOX consists of NOX1, NOX2, NOX3, NOX4, NOX5, DUOX1, and DUOX2, which are the major sources of ROS. To date, ROS signaling has been recognized to have an important effect on brain insults, including BK-induced inflammation [49]. Previously, we have also shown that NOX-2/ROS signaling displays a significant effect on MMP-9 upregulation in RBA-1 cells challenged with BK [9]. Several isoforms of NOX have been reported to participate in MMP-9 upregulation associated with numerous diseases, such as cancer, ischemic injury, and hypertension [50,51,52,53]. In this study, we made efforts to investigate whether other isoforms of NOX, such as NOX1, NOX4, and subunit p47^phox^, might affect MMP-9 expression induced by BK. Using siRNAs transfection to knock down the levels of specific isoforms, we found that downregulation of NOX1, NOX2, and subunit p47 ^phox^ is able to abrogate MMP-9 upregulation induced by BK except downregulation of NOX4. These findings suggested that NOX1, NOX2, and p47^phox^ participate in MMP-9 expression induced by BK. A study reported that isorhamnetin can dose-dependently inhibit NOX activity in aortic smooth muscle cells [54]. However, Lee et al. revealed that in H9c2 cells challenged with miconazole, Rhamnetin could reduce ROS production, but did not attenuate the expression of NOX family members [25]. This discrepancy could be due to differences in cell types and experimental conditions. It is worth verifying whether RNT possesses the inhibitory effect on NOX activity in RBA-1 cells in the future. Additionally, our findings also discovered that RNT pretreatment could decrease ROS accumulation, which is consistent with the aforementioned study [25]. Moreover, pretreatment with either PKCδ inhibitor rottlerin or NOX inhibitor DPI could also inhibit ROS generation, implying that PKCδ and NOX are upstream components of ROS in BK-induced responses. Further, to ensure whether NOX/ROS signaling contributes to BK-stimulated cell migration, we employed p47^phox^ inhibitor APO and NOX inhibitor DPI as well as ROS scavengers edaravone and NAC. Our results showed that pretreatment with these NOX inhibitors and ROS scavengers efficiently decreased BK-induced cell migration. We provided evidence supporting that the inhibitory effect of RNT on MMP-9 upregulation and cell migration induced by BK is, at least in part, achieved via blocking PKCδ-dependent NOX/ROS signaling.

Mitogen-activated protein kinase (MAPK)s components, including p38 MAPK, ERK1/2, and JNK1/2, are common intermediate downstream signalings of NOX/ROS in oxidative stress-associated inflammatory reactions. The activation of ERK1/2 signaling has been revealed to cause cell migration and proliferation [55,56]. BK has been unveiled to activate ERK1/2 and cause pathogenesis in various models of diseases [57,58,59]. Our previous report also revealed that ERK1/2 activation is involved in the upregulation of MMP-9 expression induced by BK [7]. The present finding is compatible with the previous report. In addition, our recent study showed that a small molecule of Nrf2 activator RTA 408 reduces MMP-9 expression stimulated by IL-1β via suppressing ERK1/2 pathway [60]. Several studies also indicated that isorhamnetin, a derivative of rhamnetin, suppresses the ERK1/2 pathway to process its biological effects, such as anti-oxidative stress, inhibiting tumor invasion and migration, and blocking epithelial-to-mesenchymal transition [23,24,61]. Thus, we are interested in investigating whether the RNT attenuates BK-induced cell migration and MMP-9 upregulation through inhibition of the ERK1/2 pathway. Our findings supported that RNT application, as U0126 as well, could inhibit ERK1/2 activation induced by BK, which is consistent with previous reports. We provided further evidence that in RBA-1 cells, RNT exerts its action specifically through blocking the ERK1/2 pathway to reduce MMP-9 expression and cell migration challenged with BK.

NF-κB is a protein complex consisting of several family members, including NF-κB2, RelB, c-Rel, NF-κB1, and p65, which are involved in transcription control of DNA, particularly in response to inflammation. Among these components, the p65 signaling is mainly activated in the event of stress or inflammation induced by ROS, IL-1β, BK, and LPS [33,62]. Several studies have reported that NF-κB p65 is associated with MMP-9 overexpression in a variety of cells or tissues stimulated by various pro-inflammatory mediators, including BK [7,63,64]. Therefore, NF-κB p65 signaling plays a pivotal role in neurological inflammation [65]. Our previous study indicated that ERK1/2-dependent NF-κB activity participates in MMP-9 expression induced by BK [8]. Thus, we explored whether, in RBA-1 cells stimulated by BK, the PKCδ/NOX/ROS/ERK1/2 signaling pathway could activate transcription factor NF-κB p65 activity to enhance the expression of MMP-9, associated with cell migration. Firstly, we applied the NF-κB inhibitor Bay11-7082 to pretreat cells, leading to reduction of MMP-9 protein level, MMP-9 promoter activity, and *mmp-9* gene expression stimulated by BK. Further, these findings were ascertained in RBA-1 cells by using siRNA transfection to knock down the p65 protein level and attenuate the expression of MMP-9 during the BK challenge. Next, we pretreated the cells with specific inhibitors of MEK1/2 (U0126) and NF-κB (Bay11-7082) and then determined the phosphorylation levels of NF-κB p65 stimulated by BK. We found that these two inhibitors significantly inhibited phosphorylation of NF-κB p65. Then, ChIP assay was used to examine the promoter binding activity of NF-κB in RBA-1 cells challenged with BK. Based on our data, BK enhanced p65 binding with the promoter of MMP-9 which was blocked by pretreatment with either Bay11-7082 or U0126. These findings suggested that NF-κB p65 participates in BK-induced responses, which is a downstream component of the PKCδ/NOX/ROS/ERK1/2 signaling pathway. According to previous studies, quercetin and isorhamnetin’s anti-inflammatory mechanisms are mediated through the inhibition of NF-κB activation [66]. To dissect whether the NF-κB activity is involved in the inhibitory effect of RNT on BK-induced responses in RRAB-1 cells, firstly, we pretreated the cells with RNT and then determined the phosphorylation state of NF-κB p65 stimulated by BK. Our data demonstrated that RNT inhibited phosphorylation of NF-κB p65. In addition, BK-enhanced promoter binding activity of NF-κB was blocked by RNT pretreatment, indicating that in RBA-1 cells, RNT could attenuate BK-stimulated NF-κB p65 binding activity with MMP-9 promoter element. These results suggested that the inhibitory effect of RNT on NF-κB activity is involved in the reduction of BK-induced MMP-9 expression and cell migration in RBA-1 cells. In the regulation of MMP-9 expression in RBA-1 cells, activator protein 1 (AP-1) has been unveiled as a key transcription factor [67]. Whether the inhibitory effects of RNT on MMP-9 expression are caused by suppressing AP-1 activity will be further explored in the future.

## 4. Materials and Methods

### 4.1. Materials

Enhanced chemiluminescence (ECL) detection system and Hybond C membrane were purchased from GE Healthcare Biosciences (Buckinghamshire, UK). Fetal bovine serum (FBS), Dulbecco’s modified Eagle medium (DMEM)/Ham’s nutrient mixture F-12 (F-12), and siRNAs for NOX1 (RSS300165), NOX2 (RSS330363), NOX4 (RSS331680), p47^phox^ (RSS300253), NF-κB p65 (RSS317830), and PKCδ (RSS301176) were purchased from Invitrogen (Carlsbad, CA, USA). Anti-phospho-p42/p44 MAPK (#9101), anti-NF-κB p65 (#8242), anti-phospho-p65 NF-κB (#3031), anti-Akt (#9916), and anti-phospho-PKCδ (#9374) antibodies were purchased from Cell Signaling (Danvers, MA, USA). Anti-glyceraldehyde-3-phosphate dehydrogenase (GAPDH) antibody (#MCA-ID4) was purchased from EnCor (Gainesville, FL). Anti-phospho-p47^phox^ (A1171) and anti-p47^phox^ (R12-3284) antibodies were purchased from Assay Biotech (Fremont, CA, USA). Anti-ERK1 (sc-271270) and anti-ERK2 (sc-1647) antibodies were from Santa Cruz (Santa Cruz, CA, USA). Anti-NOX1 (ab131088), anti-NOX2/gp91^phox^ (ab129068), and anti-NOX4 (ab133303) antibodies were purchased from Abcam (Cambridge, UK). All primary antibodies were diluted at 1:1000 in phosphate-buffered saline (PBS) with 1% bovine serum albumin (BSA). Edaravone was purchased from Tocris Bioscience (Bristol, UK). Diphenyleneiodonium (DPI), apocynin (APO), N-acetyl cysteine (NAC), U0126, BAY11-7082, Ro31-8220, and rottlerin were purchased from Biomol (Plymouth Meeting, PA, USA). Dihydroethidium (DHE) and 2′,7′-dichlorodihydrofluorescein diacetate (H_2_DCFDA) were purchased from Thermo Fisher Scientific (Waltham, MA, USA). Sodium dodecyl sulfate-polyacrylamide gel electrophoresis (SDS-PAGE) reagents were purchased from MDBio Inc (Taipei, Taiwan). A bicinchoninic acid (BCA) protein assay reagent was purchased from Pierce (Rockford, IL, USA). Rhamnetin was purchased from Cayman Chemicals (Ann Arbor, MI, USA). [2,3-bis-(2-methoxy-4-nitro-5-sulfophenyl)-2H-tetrazolium-5-carboxanilide] (XTT) assay kit, bradykinin (BK), dimethyl sulfoxide (DMSO), enzymes, TRIzol, and other chemicals were purchased from Sigma (St. Louis, MO, USA).

### 4.2. Cell Culture and Treatment

RBA-1 cell line was kindly provided by Professor T.C. Jou from the Institute of Neuroscience, National Yang Ming University (Taipei, Taiwan) [68]. The purity was confirmed by an astrocyte-specific marker, glial fibrillary acid protein (GFAP) staining, which reached a 95% positive rate [11]. The cells from passages 4 to 35 were used in this study. Before exposure to BK, the cells were pretreated with DMSO, RNT, or other inhibitors for 1 h.

### 4.3. Protein Preparation and Western Blotting

The cells were washed with ice-cold PBS and added with SDS-loading buffer (0.1 M Tris-HCl of pH 6.8, 1% SDS, 5% glycerol, 2.5% β-mercaptoethanol, and 0.02% bromophenol blue), as described previously [69]. After being heated for 15 min at 95 °C, the denatured proteins were vibrated for 15 s. Proteins were separated by SDS-PAGE and transferred onto Hybond-C membranes. The nitrocellulose membranes were incubated with 5% (*w/v*) BSA in Tween−Tris-buffered saline (TTBS) (Tris-HCl 50 mM, NaCl 150 mM, 0.05% (*w/v*) Tween 20, pH 7.4 at room temperature for 1 h). Then, protein level was detected by primary antibodies diluted at 1:1000 in TTBS overnight at 4 °C and a secondary horseradish peroxidase-conjugated antibody diluted at 1:1500 after being washed with TTBS four times for 10 min each. An anti-GAPDH antibody was used as an internal control. Following the secondary antibody incubation, membranes were washed comprehensively with TTBS. Enhanced chemiluminescence (ECL) reagents were used to detect the immunoreactive bands captured by a UVP BioSpectrum 500 Imaging System (Upland, CA, USA). UN-SCAN-IT gel software (Orem, UT, USA) was used to conduct the image densitometry analysis.

### 4.4. MMP Gelatin Zymography

When the inhibitors were used, the cells were pretreated with one of them for 1 h before BK exposure. Growth-arrested RBA-1 cells were incubated with BK for the indicated time intervals. As described previously [60], to remove the cells and debris, the culture media were collected and centrifuged at 1000× *g* for 10 min at 4 °C. Following centrifuging, the samples were electrophoretically separated on 10% SDS-PAGE copolymerized with 1 mg/mL gelatin (Sigma-Aldrich, St. Louis, MS, USA) under non-reducing conditions. The gels were washed twice with 2.5% Triton X-100 to remove SDS, then incubated with a developing buffer containing 50 mM Tris, 40 mM HCl, 200 mM NaCl, 5 mM CaCl_2_, and 0.02% Brij-35 at 37 °C for 72 h. A staining buffer containing 30% methanol, 10% acetic acid, and 0.5% *w/v* Coomassie Blue R-250 was used to stain the gels for 1 h and followed by being de-stained to visualize the gelatinolytic bands (MMP-9) on a dark blue background. A mixed human MMP-2 and MMP-9 (Chemicon, Temecula, CA, USA) were used for gelatinase standards. Only pro-form zymogens were quantified because cleaved MMPs are not reliably detected.

### 4.5. Total RNA Extraction, and Real-Time PCR Analysis

Total RNA from RBA-1 cells was extracted [69]. The cDNA obtained from 0.5 μg of total RNA was used as a template for PCR amplification. Based on GenBank entries for rat *MMP-9* and *GAPDH,* specific primers were designed. The following primers were used for the amplification reaction:

*GAPDH*:

Sense: 5′-AACTTTGGCATCGTGGAAGG-3′,

Antisense: 5′-GTGGATGCAGGGATGATGTTC-3′,

Probe: 5′-TGACCACAGTCCATGCCATCACTGC-3′-TAMRA;

*MMP-9*:

Sense: 5′-AGTTTGGTGTCGCGGAGCAC-3′,

Antisense: 5′-TACATGAGCGCTTCCGGCAC-3′,

Probe: 5′-CGCTCTGCATTTCTTCAAGGACGGT-3′-TAMRA.

Real-time PCR was performed with the TaqMan Gene Expression assays of Kapa Probe Fast qPCR Kit Master Mix Universal (KK4705; KAPA Biosystems, Wilmington, MA, USA) and StepOnePlus™ Real-Time PCR System (Thermo Scientific—Applied Biosystems, Foster City, CA, USA). Relative gene expression levels were determined by the ΔΔCt method, where Ct represented the threshold cycle. The levels of MMP-9 expression were quantified by standardization to the GAPDH expression. All experiments were performed in triplicate.

### 4.6. Transient siRNA Transfection

RBA-1 cells (2 × 10^5^/mL) were seeded on 6-well plates and allowed to grow until reaching about 70% confluence. As described previously [39], before transfection, the cells were washed once with PBS and incubated with 1 mL/well of DMEM/F-12 with 5% FBS. The transient transfection of siRNA was carried out using Genmute transfection reagent (SignaGen Laboratories, Rockville, MD, USA). Sequentially, the transfection reagent complexes containing a final concentration of 50 nM siRNA were dropped to each well and then incubated for 5 h at 37 °C. The cells were replaced with fresh DMEM/F-12 with 5% FBS for an additional 8 h. Before exposure to BK, the cells were washed twice with PBS and then retained in serum-free DMEM/F-12 for 16 h.

### 4.7. Rat MMP-9 Promoter Reporter Gene Assay

To construct MMP-9-luc plasmid containing the luciferase reporter system, the upstream region (−1280 to +108) of the rat MMP-9 promoter was cloned into pGL3-basic vector (Promega, Madison, WI, USA) [70]. QIAGEN plasmid DNA preparation kits were used to prepare the plasmid. A Lipofectamine reagent was used to transfect the plasmid construct into RBA-1 cells according to the instructions of the manufacturer. As described previously [69], a plasmid containing MMP-9-luc reporter system was transiently transfected along with the pCMV-Gal plasmid coding for β-galactosidase as a control which normalized transfection efficiency. To determine the promoter activity, the cells were incubated with BK, collected in a lysis buffer (25 mM Tris-phosphate, pH 7.8, 2 mM EDTA, 1% Triton X-100, and 10% glycerol), and disrupted by sonication. Finally, luciferase activities were determined by a luciferase assay system (Abcam, Cambridge, UK) and normalized to β-gal activity.

### 4.8. Chromatin Immunoprecipitation (ChIP) Assay

In this study, RBA-1 cells were seeded onto 10 cm dishes, and ChIP assay was performed as previously described [69]. At ~80–90% confluence, the culture medium was replaced with a serum-free medium overnight. Following treatment with BK, RNT, or inhibitors, the cells were fixed with 1% formaldehyde for 15 min, followed by 1.25 M glycine for 15 min at room temperature, and washed four times with PBS containing 1 mM phenylmethylsulfonyl fluoride (PMSF) and 1% aprotinin. The cells were scraped, and the lysates were prepared with SDS-lysis buffer containing 1% SDS, 5 mM EDTA, 1 mM PMSF, 0.1 mM aprotinin, 0.1 mM leupeptin, and 50 mM Tris-HCl and sonicated at 4 °C to yield 200–300 base pairs DNA fragments. The samples were centrifuged, and the soluble chromatin was precleared by incubation with sheared salmon sperm DNA-protein agarose A at 4 °C for 30 min. Samples were then centrifuged, and the protein concentrations of the supernatant were quantified and balanced. One portion of the sample was used as DNA input control, and the remains were immunoprecipitated with an anti-p65 antibody (1:100) and protein A beads. Following incubation, the samples were washed and then eluted. The cross-linking of protein-DNA complexes was reversed by heating at 65 °C overnight. DNA fragments were purified by phenol−chloroform extraction and ethanol precipitation. The purified DNA was subjected to PCR amplification using the primers specific for the region (−606 to −327, accession no. AF148065) containing the NF-κB binding elements located in the MMP-9 promoter region. The primers included a sense primer: 5′-AAGGAGTCAGCCTGCTGGGG-3′ and an antisense primer: 5′-CTAGTCCTAGGTCTGAAGGC-3′. Real-time-PCR was performed on a StepOnePlus™ real-time PCR system (Applied Biosystems, Foster City, CA, USA) using a Luna Universal qPCR Master Mix (M3003; New England BioLabs, Ipswich, MA, USA).

### 4.9. Intracellular ROS Measurement

The levels of intracellular ROS generation were determined using a fluorescent probe H_2_DCF-DA, as described previously [69]. RBA-1 cells were washed with warm PBS and incubated with 10 μM H_2_DCF-DA at 37 °C for 30 min. Subsequently, the cells were washed twice with PBS and incubated with fresh PBS. The fluorescence intensity (relative fluorescence units) was measured at 485 nm excitation and 530 nm emission using a fluorescence microplate reader (SynergyH1 Hybrid Reader, BioTek, Chittenden, VT, USA).

### 4.10. Cell Migration Assay

The cell migration assay was conducted as described previously [60]. Briefly, RBA-1 cells were cultured in 6-well culture plates, grown to confluence, and then starved in serum-free media for 24 h. The cells were scratched in the center of the well with a pipette tip. Before BK application, the cells were pretreated with inhibitors for 1 h. Migratory cells were observed with a digital camera coupled with a light microscope (Olympus, Japan). The data were summarized from three individual experiments.

### 4.11. Statistical Analysis

GraphPad Prism Program (GraphPad, San Diego, CA, USA) was adopted to analyze all the data presented as mean ± SEM. One-way ANOVA followed by Tukey’s post hoc test was used to test statistical differences between individual groups. A value of *p* < 0.05 is considered statistically significant.

## 5. Conclusions

MMP-9 upregulation during BK exposure via a PKCδ/NOX/ROS/ERK1/2-dependent NF-κB transcription activity could lead to astrocyte migration. Moreover, the present results demonstrated that in RBA-1 cells, the inhibitory effects of RNT on the expression of MMP-9 and cell migration induced by BK are attributed to its ability to suppress NF-κB transcription activity mediated through a PKCδ/NOX/ROS/ERK1/2 signaling pathway. Thus, RNT may be a good candidate in the treatment of brain inflammation associated with BK and MMP-9 overexpression. This study is worthy to be expanded by an in vivo experiment to confirm its therapeutic effects. In conclusion, our findings add new insights into the effects of RNT on anti-inflammation and its promising utilization in brain inflammatory diseases.

## Data Availability

The data presented in this study are available on request from the corresponding author.
